# Comparing Classroom Instruction to Individual Instruction as an Approach to Teach Avatar-Based Patient Monitoring With Visual Patient: Simulation Study

**DOI:** 10.2196/17922

**Published:** 2020-04-23

**Authors:** Julian Rössler, Alexander Kaserer, Benjamin Albiez, Julia Braun, Jan Breckwoldt, Donat Rudolf Spahn, Christoph Nöthiger, David Werner Tscholl

**Affiliations:** 1 University Hospital Zurich Zurich Switzerland; 2 Biostatistics and Prevention Institute Departments of Epidemiology and Biostatistics University of Zurich Zurich Switzerland

**Keywords:** avatar, computer-assisted, diagnosis

## Abstract

**Background:**

Visual Patient is an avatar-based alternative to standard patient monitor displays that significantly improves the perception of vital signs. Implementation of this technology in larger organizations would require it to be teachable by brief class instruction to large groups of professionals. Therefore, our study aimed to investigate the efficacy of such a large-scale introduction to Visual Patient.

**Objective:**

In this study, we aimed to compare 2 different educational methods, one-on-one instruction and class instruction, for training anesthesia providers in avatar-based patient monitoring.

**Methods:**

We presented 42 anesthesia providers with 30 minutes of class instruction on Visual Patient (class instruction group). We further selected a historical sample of 16 participants from a previous study who each received individual instruction (individual instruction group). After the instruction, the participants were shown monitors with either conventional displays or Visual Patient displays and were asked to interpret vital signs. In the class instruction group, the participants were shown scenarios for either 3 or 10 seconds, and the numbers of correct perceptions with each technology were compared. Then, the teaching efficacy of the class instruction was compared with that of the individual instruction in the historical sample by 2-way mixed analysis of variance and mixed regression.

**Results:**

In the class instruction group, when participants were presented with the 3-second scenario, there was a statistically significant median increase in the number of perceived vital signs when the participants were shown the Visual Patient compared to when they were shown the conventional display (3 vital signs, *P*<.001; effect size –0.55). No significant difference was found for the 10-second scenarios. There was a statistically significant interaction between the teaching intervention and display technology in the number of perceived vital signs (*P*=.04; partial η^2^=.076). The mixed logistic regression model for correct vital sign perception yielded an odds ratio (OR) of 1.88 (95% CI 1.41-2.52; *P*<.001) for individual instruction compared to class instruction as well as an OR of 3.03 (95% CI 2.50-3.70; *P*<.001) for the Visual Patient compared to conventional monitoring.

**Conclusions:**

Although individual instruction on Visual Patient is slightly more effective, class instruction is a viable teaching method; thus, large-scale introduction of health care providers to this novel technology is feasible.

## Introduction

**Figure 1:**
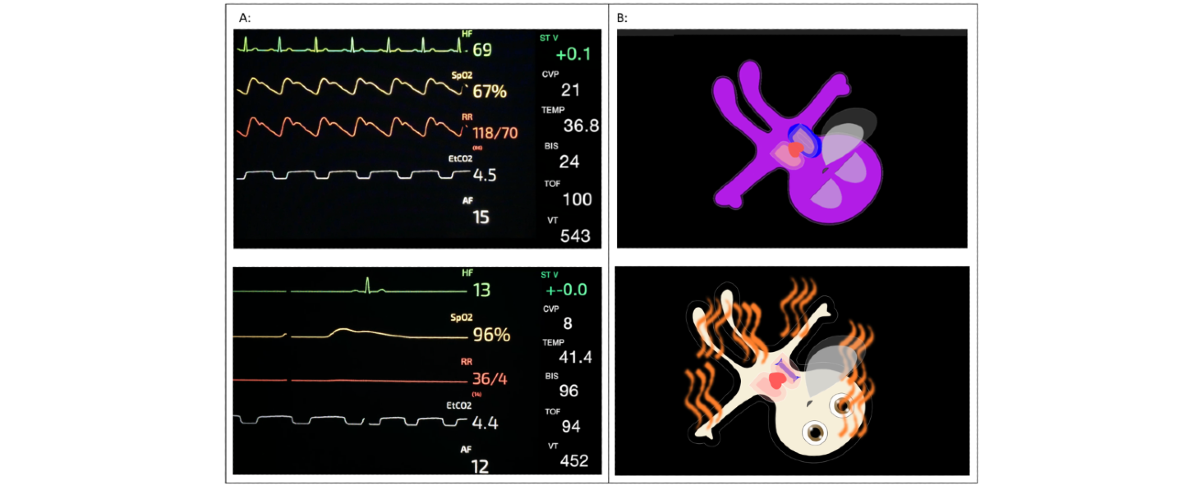
Screenshots of the presented scenarios showing conventional monitoring (A) and avatar-based monitoring with the Visual Patient (B).

**Figure 2:**
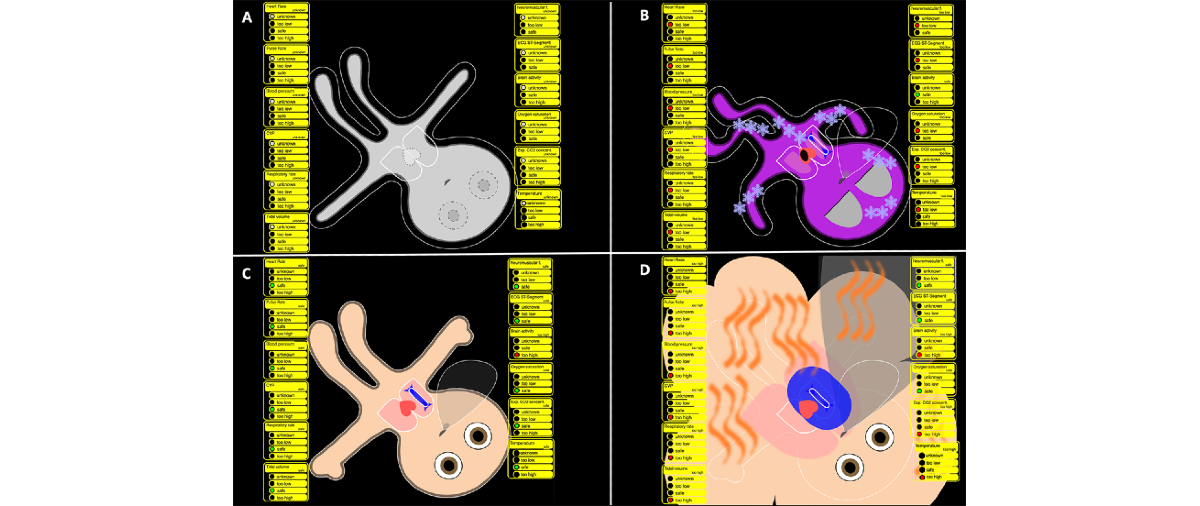
Vital sign parameters of the Visual Patient with a legend showing how each parameter is visualized. A: Visual Patient display when no vital sign data are received. B: Desaturated, hypothermic patient with ST-segment deviation. C: Visual Patient with all vital signs in a safe state and high brain activity (open eyes). D: Hypertensive, hyperthermic patient with high central line pressure.

Monitoring and continuous evaluation of vital signs by anesthesia providers is central to perioperative patient safety [1]. With 313 million surgeries performed worldwide every year, patient monitors are ubiquitous in perioperative health care [2]. However, there have been no recent substantial changes to the industry standard of displaying vital signs as numbers and curves, and some anesthesia providers report difficulties regarding this form of presentation [3]. Considering the design principles of situation awareness, Visual Patient was developed as an additional way to present vital signs [4-6]. The Visual Patient displays vital signs by modification of an animated avatar (Figure 1, Multimedia Appendix 1). The avatar, which corresponds to the patient, can display 11 vital signs; for example, it pulsates with different intensities and frequencies, breathes, and changes color on desaturation (Figure 2, Multimedia Appendix 1). Tscholl and colleagues were able to show that after briefly seeing a display of the Visual Patient, anesthesia providers were able to recall more vital signs than with conventional monitoring. They further reported improved confidence and reduced cognitive effort [4,7]. This may help healthcare providers gain situation awareness more efficiently and may increase patient safety [8-11]. However, the implementation of this technology may be difficult, as conventional monitoring is well known and established. Feasibility of Visual Patient training for widespread implementation would require the training to be deliverable to multiple participants at once, short in duration (eg, 30 minutes), and suitable for large auditoriums.

We designed a simulation study where participants who had no previous experience with Visual Patient underwent either individual or classroom-based instruction and were then asked to interpret conventional displays and avatar-based Visual Patient displays. We hypothesized that the 2 instruction methods would be comparable in efficacy as an introduction to avatar-based monitoring with Visual Patient.

## Methods

### Participants and Trial Design

On January 19, 2019, 42 nurse anesthetists were recruited to the classroom instruction group. Since the study did not include any real patient data or any human material, the research project did not fall into the scope of the Human Research Act and did not require ethics committee approval. However, we obtained written consent from all participants to use the collected data for scientific purposes.

We delivered a 30-minute plenary presentation to all participants in the classroom instruction group. The presentation included an introduction to the concept and technology of Visual Patient as well as an educational video on how the system is used (supplementary video 1 in Multimedia Appendix 1). Subsequently, the participants were shown 4 scenarios in a randomized order. In 2 scenarios, vital signs were presented with the Visual Patient, and in the other 2 scenarios, the vital signs were presented as in conventional monitoring. The display scenarios were projected on a screen for either 3 or 10 seconds, after which the screen was blacked out. After each scenario, the participants were asked to rate every presented vital sign as normal, abnormal, or not perceived. Data collection was simultaneous for all participants, as each individual’s desk was equipped with an iPad (Apple, Inc) containing a questionnaire (iSURVEY, Harvest Your Data) for the participants to complete [12].

The individual instruction group consisted of a selected sample from a previously published study on Visual Patient [4]. We selected 16 participants who were shown the same scenarios as the class instruction group. The methodology of this study is described in the previous publication. In brief, the data collection was similar for these participants, except that each participant was individually introduced to the Visual Patient followed by presentation of the scenarios to the participant alone.

### Outcomes

To assess the educational success of class instruction on the Visual Patient technology, each rating of a vital sign was graded as correct or incorrect. This enabled us to compare the correct and incorrect perceptions of the vital signs displayed with both technologies.

At the end of the study, participants rated their introduction to the Visual Patient on a 5-point Likert scale (1=insufficient, 2=inadequate, 3=O.K., 4=good, and 5=very good).

### Statistical Analysis

Data are provided as medians and interquartile ranges (IQR) regardless of normality or estimated marginal means for linear models. Normality was assessed with the Shapiro-Wilks test and visual inspection of quantile-quantile plots of dependent variables. Binary variables are presented as frequencies with percentages. The Wilcoxon signed-rank test was conducted to determine the effects of the Visual Patient display on the ability to correctly perceive vital signs after seeing the display for either 3 or 10 seconds. The different scores were approximately symmetrically distributed, as assessed by box plots. For both scenarios, post hoc descriptive graphs were created detailing whether each vital sign was perceived correctly, incorrectly, or not at all.

To compare the effects of classroom instruction and individual instruction, 2-way mixed analysis of variance (ANOVA) was calculated with the factors of display technology (within-subject) and instruction method (between-subject). There was a single outlier, as assessed by inspection of a box plot for values greater than 1.5 box lengths from the edge of the box. As the studentized residual for this outlier was only 3.06, it was retained in the analysis. Homogeneity was observed for variances (*P*>.05) and covariances (*P*>.001), as assessed by the Levene test of homogeneity of variances and the Box M test, respectively.

We fitted a mixed logistic regression model for the correct perception of vital signs with a random intercept for each participant. The model included the instruction variable, which denoted whether the participant received individual instruction or classroom instruction. We additionally adjusted for the display mode (Visual Patient vs conventional monitoring), the duration of the task (3 seconds vs 10 seconds), and the previous experience of the participants.

Analyses were conducted in SPSS 25 (IBM Corporation) and R version 3.6.1 (R Foundation for Statistical Computing). Figures were created using GraphPad Prism 8.1.1 (GraphPad Software, Inc). As group differences were calculated separately for both scenarios, a Bonferroni adjusted *P* value <.025 was considered to indicate statistical significance.

### Availability of Data and Material

The data sets used and analyzed during the current study are available from the corresponding author on reasonable request.

## Results

### Participants

The 42 nurse anesthetists participating in the study reported a median professional experience of 12 years (IQR 3-31). Of the participants, 28/42 (67%) were female. As they were presented in randomized order with 2 sets, each consisting of a Visual Patient scenario and a matched conventional display scenario, 84 direct within-subject comparisons were performed. After the study, most participants in the class instruction group rated the introductory presentation as very good (20/42, 48%) or as good (13/42, 31%), whereas 9 participants did not take part in the follow-up survey.

The selected sample of 16 participants from a previous study, who received individual instruction on the Visual Patient, consisted of 8 (50%) physician anesthetists and 8 (50%) nurse anesthetists, where 11 (69%) were female. Table 1 gives an extended overview of the characteristics of the participants.

**Table 1:** Participant characteristics.

Characteristic		Class instruction group (n=42)		Individual instruction group (n=16)	
**Gender, n (%)**					
	Female		28 (67)		11 (69)
	Male		14 (33)		5 (31)
**Profession, n (%)**					
	Nurse anesthetist		42 (100)		8 (50)
	Physician anesthetist		0 (0)		8 (50)
**Experience, n (%)**					
	<1 year		2 (5)		1 (6)
	1-5 years		9 (21)		5 (31)
	>10 years		3 (7)		4 (25)
	5-10 years		17 (40)		6 (38)
	Unknown		11 (26)		0 (0)

### Perception of Vital Signs

**Figure 3:**
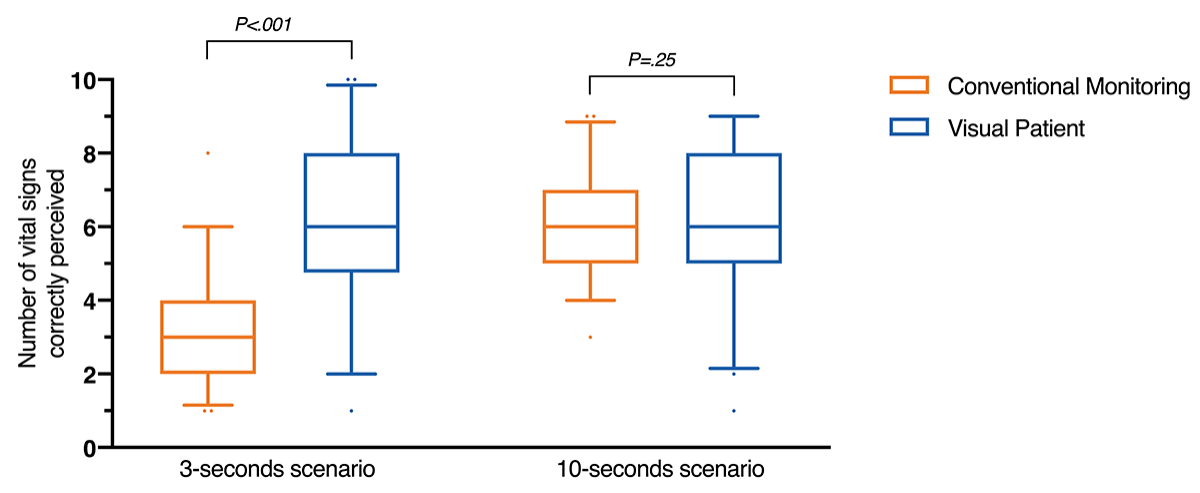
Box plots of the vital signs that were correctly perceived with both the Visual Patient and conventional monitoring. Participants were shown scenarios for either 3 or 10 seconds. Group differences were assessed by Wilcoxon signed-rank test. The whiskers indicate the 5th and 95th percentiles.

After the classroom instruction, when presented with the 3-second scenarios, participants were able to correctly perceive a median of 6 vital signs (IQR 4.8-8) with the Visual Patient and a median of 3 vital signs (IQR 2-4) with the conventional monitoring display. The Wilcoxon signed-rank test determined a significant median increase in the perception of vital signs (3) when participants were shown the Visual Patient compared to when they were shown the conventional display (z=–5.0; *P*<.001) with a large effect size of –0.55 (Figure 3).

When the 10-second scenarios were shown after the class instruction, participants were able to correctly perceive a median of 6 vital signs (IQR 5-8) with the Visual Patient and a median of 6 vital signs (IQR 5-7) with the conventional monitoring display. Thus, there was no statistically significant median increase in the perception of vital signs (z=–1.2; *P=*.25) as determined by Wilcoxon signed-rank test (Figure 3).

**Figure 4:**
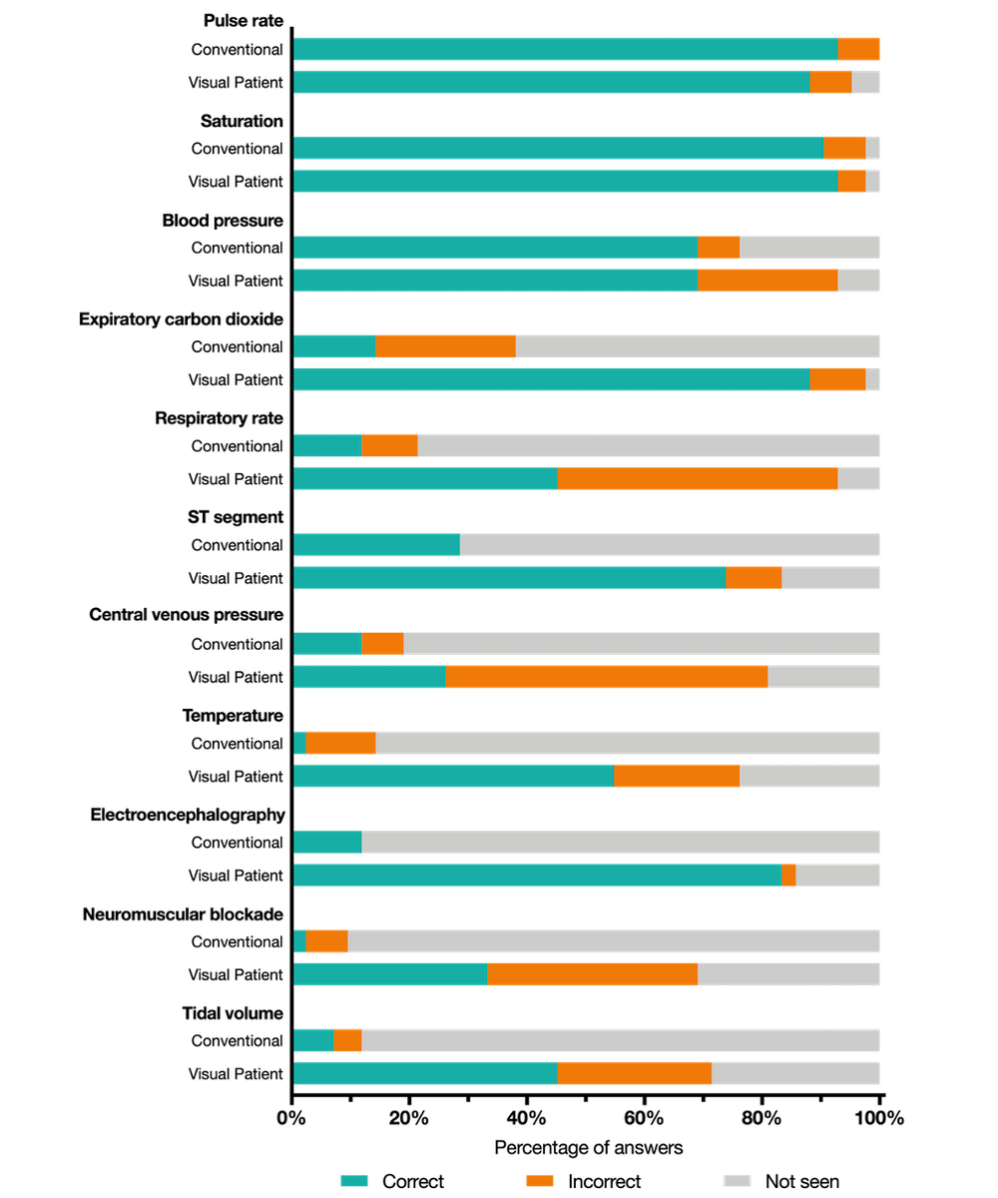
Stacked bar graph indicating the perception of presented vital signs after the 3-second scenario. Percentages were calculated from the 4 possible answers to each vital sign: too high, normal, too low, and did not perceive. Depending on the presented scenario, the answers were rated as correct, incorrect, or not seen.

Vital sign–specific descriptive analysis in the class instruction group showed that in the 3-second scenarios, nearly all participants were able to correctly perceive the pulse rate and oxygen saturation. Furthermore, most participants correctly recalled the blood pressure. The overall group difference was largest within the other parameters, as shown in Figure 4. Moreover, with the Visual Patient, the correct perceptions increased, but the incorrect perceptions also increased; therefore, the number of unperceived vital signs decreased (Figure 4, Multimedia Appendix 2). For the 10-second scenarios, vital sign–specific descriptive analysis is available in Multimedia Appendix 2.

### Effects of the Instruction Method

The 2-way mixed ANOVA indicated a statistically significant interaction between the teaching intervention and display technology for perceived vital signs (F_1,56_ 4.61; *P*=.04; partial η^2^=.076). Post-hoc univariate analysis yielded a statistically significant difference between the 2 teaching interventions for the Visual Patient (F_1,56_ 14.42; *P*<.001; partial η^2^=.205) but not for conventional monitoring (F_1,56_ 3.06; *P*=.09; partial η^2^=.052).

**Figure 5:**
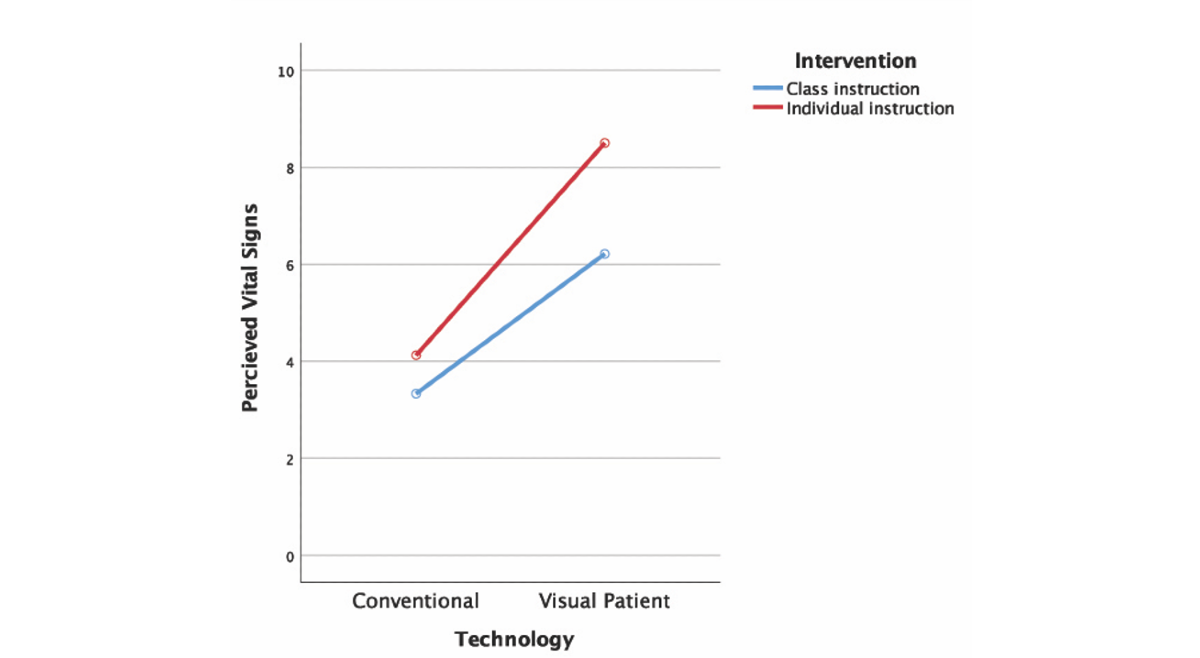
Marginal means of the perceived vital signs by the 2 instruction groups estimated by 2-way mixed ANOVA.

In the classroom instruction group, the estimated marginal means of the perceived vital signs increased from 3.3 (95% CI 2.9-3.8) with conventional monitoring to 6.2 (95% CI 5.6-6.8) with the Visual Patient. In the individual instruction group, the estimated marginal means of the perceived vital signs increased from 4.1 (95% CI 3.4-4.9) with conventional monitoring to 8.5 (95% CI 7.5-9.5) with the Visual Patient. As shown in Figure 5, this resulted in a mean difference of 2.3 between the number of vital signs perceived with the Visual Patient in the 2 instruction groups (95% CI 1.1-3.5; *P*<.001).

### Mixed Logistic Regression

The mixed logistic regression model showed evidence of a difference between the teaching modes in favor of individual instruction, yielding OR 1.88 (95% CI 1.41-2.52; *P*<.001) for correct vital sign perception after individual instruction. Moreover, the model displayed very strong evidence for the superiority of Visual Patient, with OR 3.03 (95% CI 2.50-3.70; *P*<.001) for correct vital sign perception with the Visual Patient (Table 2).

**Table 2:** Mixed logistic regression for correct perception of vital signs with the random intercept for each participant.

Variable		OR^a^ (95% CI)	*P* value
**Teaching mode**			
	Class	Reference	N/A^b^
	Individual	1.88 (1.41-2.52)	<.001
**Display technology**			
	Conventional	Reference	N/A
	Visual Patient	3.03 (2.50-3.70)	<.001
**Scenario duration**			
	3 seconds	Reference	N/A
	10 seconds	2.31 (1.91-2.80)	<.001
**Experience**			
	<1 year	Reference	N/A
	1-5 years	1.21 (0.67-2.16)	.53
	5-10 years	1.01 (0.55-1.88)	.97
	>10 years	0.86 (0.49-1.50)	.59

^a^OR: odds ratio.

^b^Not applicable.

## Discussion

### Principal Findings

Avatar-based patient monitoring is an alternative way to display vital signs. It can facilitate perception, reduce mental workload, and increase situation awareness [4,13]. This technology is generally well received by users and thought to be easy to learn; however, to implement it in larger health care systems, it must be trainable via class instruction [14].

In this study, we presented 42 anesthesia providers with 0.5 hours of class instruction on Visual Patient. Afterward, they were shown monitors with either conventional displays or Visual Patient displays and asked to interpret vital signs. If the participants saw the scenarios for 3 seconds, they were able to perceive significantly more vital signs with the Visual Patient. Further, the calculated effect size of the Visual Patient on correct perceptions was large (–0.55). No significant difference was found for the 10-second scenarios. These results are similar to those of a previous study by our research group on Visual Patient, where more vital signs were perceived with the Visual Patient after both 3 and 10 seconds [4]. However, in this study, the median difference was also less for the 10-second scenarios [4]. In the previous study, instruction was individual. To compare the efficacy of both instruction methods, we therefore compared the current sample with a selected historical sample from the previous study. While both instruction methods were successful, individual instruction yielded slightly better results.

In daily clinical practice, the superiority of the Visual Patient when seeing a monitor for 3 seconds may already be very relevant. It has been shown that anesthesia providers tend to look at patient monitors in short glances [9]. These glances become more frequent during critical situations, where vital signs can change rapidly and many can change at once [9]. In these cases, the median increase of 3 more vital signs perceived with the Visual Patient may make a crucial difference.

**Figure 6:**
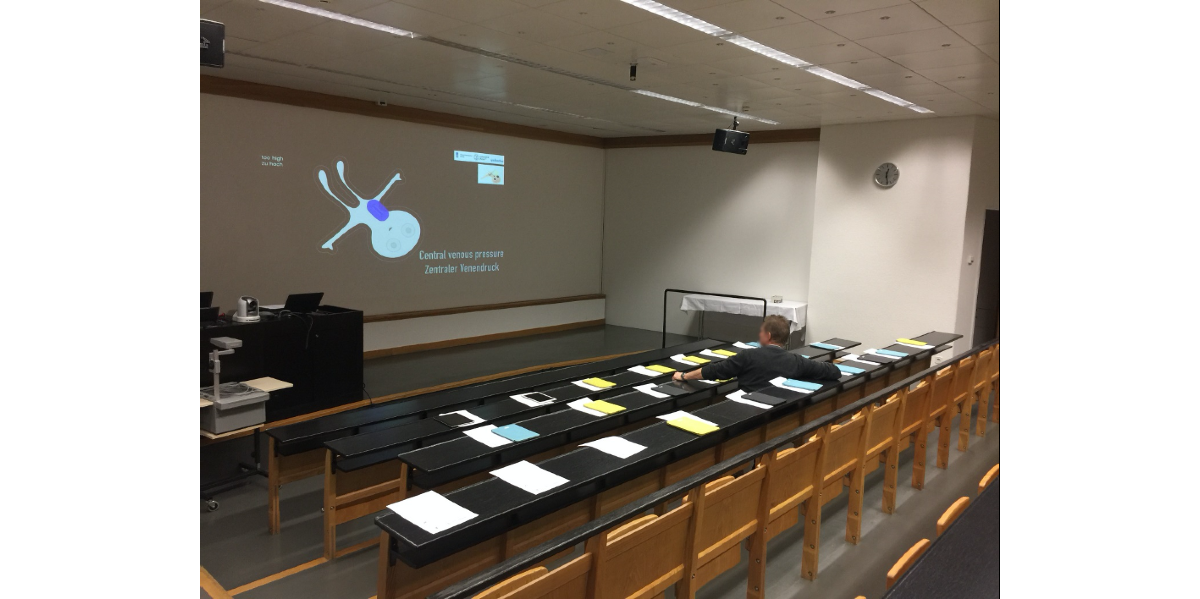
The auditorium in which the introduction to the Visual Patient was conducted.

Participants were introduced to the Visual Patient according to our prespecified necessary criteria for general implementation. The teaching was conducted with 30 minutes of plenary classroom instruction (Figure 6), which was well received by the participants. The replication of results from previous studies, where each participant was introduced to the Visual Patient in a one-on-one setting, shows the feasibility of large-scale teaching. If avatar-based monitoring is implemented in health care systems or single hospitals, one-on-one teaching of each employee will not be practical. Employees will need to be trained to use the technology in a setting similar to that in our study [15]. Alternatively, e-learning may be considered or even no instruction at all, as the Visual Patient technology is generally perceived as intuitive to understand [14]. Animated avatars have been used to provide visual support in the education of patients with sensory impairment in the form of assistive computer vision [16,17] as well as in the education of children with autism, where an avatar can display emotions and support affective learning [18,19]. As an avatar is a manifestation of self and reflects already known images or movements in simplified ways, the avatar itself can be used as an educational tool [20]. Therefore, implementation of avatars without instruction may be a subject of future study.

Post hoc analysis of perception of specific vital signs showed that with both technologies, the pulse rate and oxygen saturation were nearly always perceived correctly; also, the blood pressure was perceived correctly in approximately 69% of cases. In conventional monitoring displays, these figures are often the largest displayed and are thus easily perceived. The vital signs with more pronounced differences (expiratory carbon dioxide, respiratory rate, ST segment, central venous pressure, temperature, electroencephalography, neuromuscular blockade, and tidal volume) may be displayed in smaller sizes or in less prominent places. One advantage of the Visual Patient is that all vital signs are displayed in close proximity to each other and sometimes repeatedly (eg, the respiratory rate can be deduced from the lung movement or the expired “gas bubble”). This is supported by an eye-tracking study on Visual Patient, which showed that participants were able to visually fixate on more vital signs with Visual Patient than with conventional monitoring [21]. The close proximity further facilitates perception by peripheral vision [22]. Another advantage of Visual Patient is that due to the way the vital signs are displayed, parallel acquisition of information is possible. For example, users can recognize the pulsation frequency, color, and shape of an object in a single glance. To do the same in conventional number-based and waveform-based patient monitoring, users must read several numbers in several glances. [23]

Although the introduction to Visual Patient seemed to be sufficient and the monitoring capability improved, further progress may be possible with more detailed teaching or continued clinical use. The vital sign–based analysis showed that while correct perception of vital signs increased with the Visual Patient, incorrect perception increased as well. This may be due either to the design of these parameters or to inexperience with the Visual Patient. More detailed user perception studies are required to evaluate this result; however, it is more likely to be due to inexperience. The Visual Patient parameters and their display were calibrated using a Delphi process and generally show high interrater reliability, with a previously reported Fleiss kappa >.94 [4]. Participants seemed to be able to perceive these vital signs, as corroborated by the eye-tracking study [21]; however, their knowledge of Visual Patient may still have been insufficient to correctly interpret them. This implies that with further clinical use and practice, Visual Patient will yield even better situation awareness.

### Strengths and Limitations

This study had some limitations. The study was simulation based; thus, translational evidence for clinical practice may be limited. Further studies in a high-fidelity simulation environment or in clinical practice are required. However, it is plausible that the effects would persist if used in a clinical setting, as the basic physiological specifications of information intake do not change. The results are in line with those of similar avatar-based monitoring systems, such as the Visual Clot, an animated blood clot that represents coagulation disorders [24]. This study also had particular strengths. The examined group was somewhat heterogenous, which increases the external validity. However, more physicians should be included in further studies. The study was not conducted in a sensory-sterile environment; both the instruction and data collection were performed with a large group, where the possible distractions are more similar to a real clinical atmosphere.

### Conclusions

Although individual instruction on the Visual Patient is slightly more effective, class instruction is a viable teaching method; this increases the feasibility of large-scale introduction of health care providers to this novel technology. This study further contributes to the growing evidence of the superiority of avatar-based monitoring to conventional monitoring in certain situations.

## Appendix

Multimedia Appendix 1 Supplementary video 1: the Visual Patient educational video shown to all participants of this study.

Multimedia Appendix 2 Supplementary tables and figures.

## Abbreviations

ANOVA: analysis of variance
IQR: interquartile range
OR: odds ratio
