# Correction: Comparing Classroom Instruction to Individual Instruction as an Approach to Teach Avatar-Based Patient Monitoring With Visual Patient: Simulation Study

**DOI:** 10.2196/24459

**Published:** 2020-10-01

**Authors:** Julian Rössler, Alexander Kaserer, Benjamin Albiez, Julia Braun, Jan Breckwoldt, Donat Rudolf Spahn, Christoph Nöthiger, David Werner Tscholl

**Affiliations:** 1 University Hospital Zurich Zurich Switzerland; 2 Biostatistics and Prevention Institute Departments of Epidemiology and Biostatistics University of Zurich Zurich Switzerland

In “Comparing Classroom Instruction to Individual Instruction as an Approach to Teach Avatar-Based Patient Monitoring With Visual Patient: Simulation Study” (JMIR Med Educ 2020;6(1):e17922) the authors noted one error.

Degree information for author Benjamin Albiez was incorrectly listed as "MD". This has been corrected to "RN, BNSc".

The correction will appear in the online version of the paper on the JMIR Publications website on October 1, 2020, together with the publication of this correction notice. Because this was made after submission to PubMed, PubMed Central, and other full-text repositories, the corrected article has also been resubmitted to those repositories.

