# Supplementary material for: Comparing Classroom Instruction to Individual Instruction as an Approach to Teach Avatar-Based Patient Monitoring With Visual Patient: Simulation Study
Source: JMIR Med Educ. 2020 Apr 23;6(1):e17922. doi: 10.2196/17922 (PMC7206517; doi:10.2196/17922)
Supplement: Multimedia Appendix 2 [file mededu_v6i1e17922_app2.docx]

Supplementary Appendix

Feasibility of a large-scale introduction to avatar-based monitoring with the Visual Patient: An experimental study.

Table of contents:

Supplementary Table 1: **Vital sign and scenario specific frequency of perceptions** – Page 2

Supplementary Figure 1: **Stacked bar graph for perception after the ten seconds scenario** – Page 3

**Supplementary Table 1.** Vital sign and scenario specific frequency of perceptions. Data is number of answers (%).

|  |  | **3 second scenarios** | | | **10 second scenarios** | | |
| --- | --- | --- | --- | --- | --- | --- | --- |
|  |  | *Correct* | *Incorrect* | *Not seen* | *Correct* | *Incorrect* | *Not seen* |
| Pulse rate | Conventional | 39 (92.9) | 3 (7.1) | 0 (0) | 35 (83.3) | 6 (14.3) | 1 (2.4) |
|  | Visual Patient | 37 (88.1) | 3 (7.1) | 2 (4.8) | 41 (97.6) | 1 (2.4) | 0 (0) |
| Oxygen saturation | Conventional | 38 (90.5) | 3 (7.1) | 1 (2.4) | 34 (81) | 8 (19) | 0 (0) |
|  | Visual Patient | 39 (92.9) | 2 (4.8) | 1 (2.4) | 39 (92.9) | 2 (4.8) | 1 (2.4) |
| Blood pressure | Conventional | 29 (69) | 3 (7.1) | 10 (23.8) | 29 (69) | 9 (21.4) | 4 (9.5) |
|  | Visual Patient | 29 (69) | 10 (23.8) | 3 (7.1) | 42 (100) | 0 (0) | 0 (0) |
| Expiratory carbon dioxide | Conventional | 6 (14.3) | 10 (23.8) | 26 (61.9) | 25 (59.5) | 12 (28.6) | 5 (11.9) |
|  | Visual Patient | 37 (88.1) | 4 (9.5) | 1 (2.4) | 29 (69) | 7 (16.7) | 6 (14.3) |
| Respiratory rate | Conventional | 5 (11.9) | 4 (9.5) | 33 (78.6) | 26 (61.9) | 6 (14.3) | 10 (23.8) |
|  | Visual Patient | 19 (45.2) | 20 (47.6) | 3 (7.1) | 24 (57.1) | 12 (28.6) | 6 (14.3) |
| ST segment | Conventional | 12 (28.6) | 0 (0) | 30 (71.4) | 14 (33.3) | 6 (14.3) | 22 (52.4) |
|  | Visual Patient | 31 (73.8) | 4 (9.5) | 7 (16.7) | 16 (38.1) | 12 (28.6) | 14 (33.3) |
| Central venous pressure | Conventional | 5 (11.9) | 3 (7.1) | 34 (81) | 10 (23.8) | 10 (23.8) | 22 (52.4) |
|  | Visual Patient | 11 (26.2) | 23 (54.8) | 8 (19) | 6 (14.3) | 11 (26.2) | 25 (59.5) |
| Temperature | Conventional | 1 (2.4) | 5 (11.9) | 36 (85.7) | 26 (61.9) | 4 (9.5) | 12 (28.6) |
|  | Visual Patient | 23 (54.8) | 9 (21.4) | 10 (23.8) | 22 (52.4) | 9 (21.4) | 11 (26.2) |
| Electroencephalography | Conventional | 5 (11.9) | 0 (0) | 37 (88.1) | 17 (40.5) | 8 (19) | 17 (40.5) |
|  | Visual Patient | 35 (83.3) | 1 (2.4) | 6 (14.3) | 16 (38.1) | 4 (9.5) | 22 (52.4) |
| Neuromuscular blockade | Conventional | 1 (2.4) | 3 (7.1) | 38 (90.5) | 24 (57.1) | 3 (7.1) | 15 (35.7) |
|  | Visual Patient | 14 (33.3) | 15 (35.7) | 13 (31) | 10 (23.8) | 7 (16.7) | 25 (59.5) |
| Tidal volume | Conventional | 3 (7.1) | 2 (4.8) | 37 (88.1) | 21 (50) | 5 (11.9) | 16 (38.1) |
|  | Visual Patient | 19 (45.2) | 11 (26.2) | 12 (28.6) | 16 (38.1) | 5 (11.9) | 21 (50) |


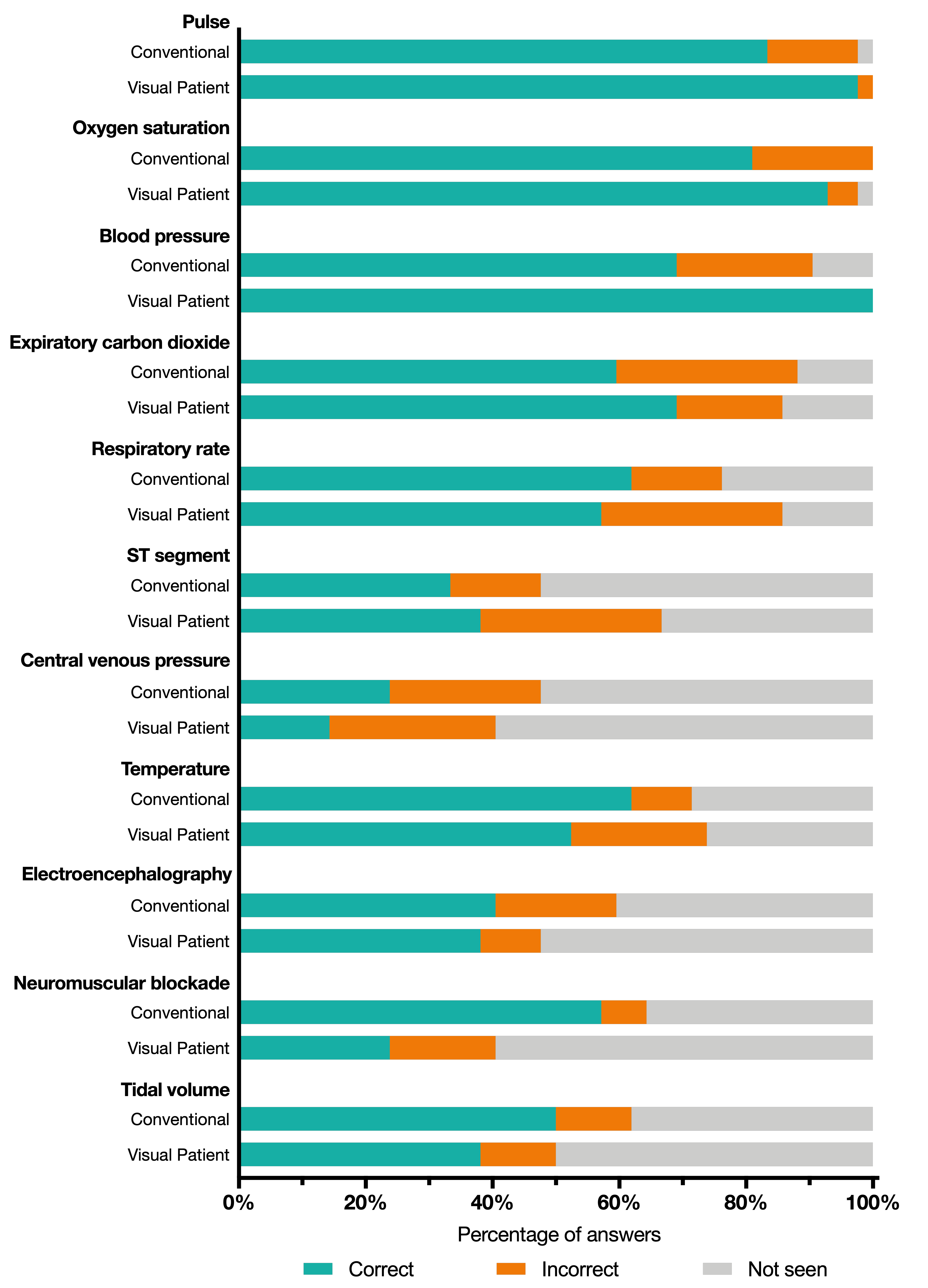
**Supplementary Figure 1.** Stacked bar graph for vital sign-based presentation of perception after the ten seconds scenario. Percentages were calculated from the four possible answers to each vital sign: “too high”, “normal”, “too low”, or “did not perceive”. Depending on the presented scenario, the answers were then rated as “correct”, “incorrect” or “not seen”.
